# Structure and Mechanism of LcpA, a Phosphotransferase That Mediates Glycosylation of a Gram-Positive Bacterial Cell Wall-Anchored Protein

**DOI:** 10.1128/mBio.01580-18

**Published:** 2019-02-19

**Authors:** Sara D. Siegel, Brendan R. Amer, Chenggang Wu, Michael R. Sawaya, Jason E. Gosschalk, Robert T. Clubb, Hung Ton-That

**Affiliations:** a Department of Microbiology and Molecular Genetics, University of Texas Health Science Center, Houston, Texas, USA; b Department of Chemistry and Biochemistry and the UCLA-DOE Institute of Genomics and Proteomics, University of California, Los Angeles, California, USA; c Division of Oral Biology and Medicine, School of Dentistry, University of California, Los Angeles, California, USA; Georgia Institute of Technology School of Biological Sciences; NIAID, NIH; University of Alabama at Birmingham

**Keywords:** LCP, X-ray crystallography, cell wall, Gram-positive bacteria, phosphotransferase, protein folding, protein glycosylation, sortase

## Abstract

The widely conserved LytR-CpsA-Psr (LCP) family of enzymes in Gram-positive bacteria is known to attach glycopolymers, including wall teichoic acid, to the cell envelope. However, it is undetermined if these enzymes are capable of catalyzing glycan attachment to surface proteins. In the actinobacterium Actinomyces oris, an LCP homolog here named LcpA is genetically linked to GspA, a glycoprotein that is covalently attached to the bacterial peptidoglycan by the housekeeping sortase SrtA. Here we show by X-ray crystallography that LcpA adopts an α-β-α structural fold, akin to the conserved LCP domain, which harbors characteristic catalytic arginine residues. Consistently, alanine substitution for these residues, R149 and R266, abrogates GspA glycosylation, leading to accumulation of an intermediate form termed GspA^LMM^, which is also observed in the *lcpA* mutant. Unlike other LCP proteins characterized to date, LcpA contains a stabilizing disulfide bond, mutations of which severely affect LcpA stability. In line with the established role of disulfide bond formation in oxidative protein folding in *A. oris*, deletion of *vkor*, coding for the thiol-disulfide oxidoreductase VKOR, also significantly reduces LcpA stability. Biochemical studies demonstrated that the recombinant LcpA enzyme possesses pyrophosphatase activity, enabling hydrolysis of diphosphate bonds. Furthermore, this recombinant enzyme, which weakly interacts with GspA in solution, catalyzes phosphotransfer to GspA^LMM^. Altogether, the findings support that *A. oris* LcpA is an archetypal LCP enzyme that glycosylates a cell wall-anchored protein, a process that may be conserved in *Actinobacteria*, given the conservation of LcpA and GspA in these high-GC-content organisms.

## Abstract (precis)

In Gram-positive bacteria, the conserved LCP family enzymes studied to date are known to attach glycopolymers, including wall teichoic acid, to the cell envelope. It is unknown if these enzymes catalyze glycosylation of surface proteins. We show here in the actinobacterium Actinomyces oris by X-ray crystallography and biochemical analyses that *A. oris* LcpA is an LCP homolog, possessing pyrophosphatase and phosphotransferase activities known to belong to LCP enzymes that require conserved catalytic Arg residues, while harboring a unique disulfide bond critical for protein stability. Importantly, LcpA mediates glycosylation of the surface protein GspA via phosphotransferase activity. Our studies provide the first experimental evidence of an archetypal LCP enzyme that promotes glycosylation of a cell wall-anchored protein in Gram-positive bacteria.

## INTRODUCTION

Glycopolymers, such as wall teichoic acids (WTAs), capsular polysaccharides, and lipoteichoic acids (LTAs), displayed on the cell envelope of Gram-positive bacteria play critical roles in cell physiology by modulating immunogenicity, host and bacterial surface interactions, protein stability, cell division, and affinity for charged molecules, including antimicrobial peptides and cations (1–4). WTAs are alditol phosphate-containing polymers that end in a disaccharide linkage unit that is attached via a phosphodiester bond to the C-6 hydroxyl group of *N*-acetylmuramic acid (MurNAc) of bacterial peptidoglycan, and LTAs are structurally related and consist of polyglycerol phosphate linked to the bacterial membrane via a diacylglycerol lipid (5). It is thought that attachment of WTAs and capsular polysaccharides to the cell envelope requires LytR-CpsA-Psr (LCP) family enzymes that are widespread in Gram-positive bacteria (6). Structural and functional studies of the LCP enzyme from Streptococcus pneumoniae, CpsA2, provided the first insight into the enzymatic mechanism. CpsA2 contains a large hydrophobic tunnel that is capped with surface-exposed arginine residues that are important for catalysis (7); serendipitously, CpsA2 cocrystallizes with octaprenyl-pyrophosphate (Opr-PP), where the isoprenyl tail is nestled within the hydrophobic pocket with the pyrophosphate head group interacting with highly conserved arginine residues within the active site. LCP proteins attach WTA to the cell by catalyzing the formation of a phosphodiester bond to link the glycan onto the MurNAc of the cell wall (8). Consistent with this notion, it has previously been demonstrated that S. pneumoniae CpsA2 and Corynebacterium glutamicum LcpA possess pyrophosphatase activity, and this is likely a characteristic of most LCP enzymes (7, 9–13). Recently, the cell wall ligase activity of the Bacillus subtilis and Staphylococcus aureus LCP enzymes has been reconstituted *in vitro* (11, 12). In addition, the structure of an S. aureus LCP enzyme in complex with a WTA precursor has been determined (11), defining the location of the peptidoglycan binding site and leading to the conclusion that LCP enzymes attach wall teichoic acids to un-cross-linked peptidoglycan chains at an early stage in cell wall synthesis.

An LCP enzyme has also been identified in the Gram-positive actinobacterium Actinomyces oris, a key colonizer of the oral cavity that plays an important role in the development of oral biofilms or dental plaque (14). The *A. oris* LCP, here referred to as LcpA, has been implicated in glycosylation of the cell wall-anchored protein GspA (15). The adjacent presence of *lcpA* and *gspA* genes in *A. oris* and genetic characterizations indicate that their protein products are functionally linked (15). Biochemical and genetic evidence supports that GspA is highly glycosylated and this glycosylation involves LcpA; the isogenic mutant strain lacking *lcpA* no longer produces high-molecular-mass glycopolymers of GspA, resulting in accumulation of intermediate forms (15). Glycosylation of GspA does not appear to occur on peptidoglycan as glycopolymers are still detected with a GspA mutant lacking a C-terminal cell wall sorting signal (CWSS) (15), which permits covalent attachment to peptidoglycan by the *A. oris* sortase (SrtA) enzyme (16). A model of GspA glycosylation involving both LcpA and SrtA has previously been proposed; as GspA is translocated across the cytoplasmic membrane by the Sec machinery, it is glycosylated by LcpA, with the glycan chain synthesized by a separate unknown pathway, and subsequently anchored to the cell wall by the housekeeping sortase SrtA (15).

While the exact nature and composition of the GspA glycans remain to be biochemically determined, it is apparent that *A. oris* LcpA represents the first example of an LCP enzyme that modifies a cell wall-anchored protein substrate. Here, we report a 2.5-Å crystal structure of *A. oris* LcpA, revealing conserved features of known LCP enzymes and unique characteristics that may be typical of actinobacterial LCP proteins. Further biochemical characterizations provide evidence that LcpA possesses pyrophosphatase activity and also functions as a phosphotransferase, catalyzing glycosylation of the cell wall-anchored protein GspA.

## RESULTS

### LcpA is the sole LCP enzyme required for GspA glycosylation in *A. oris*.

**FIG 1:**
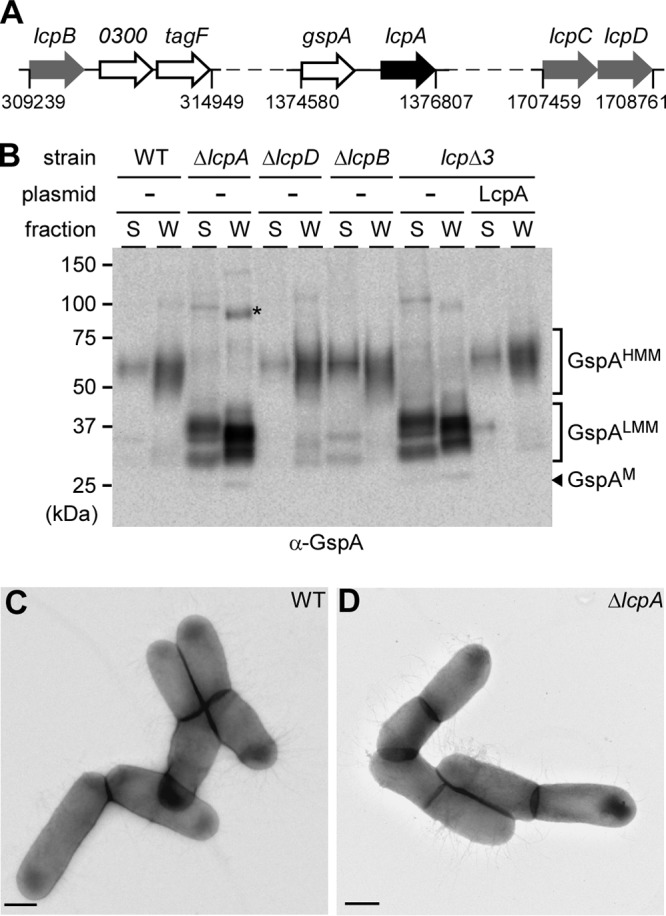
LcpA is solely responsible for GspA glycosylation. (A) Presented are gene clusters that encode four LCP proteins (LcpA to -D), with numbers indicating the nucleotide positions of *lcp* genes. (B) *A. oris* cells of indicated strains grown to early log phase were subjected to cell fractionation. Culture medium (S) and cell wall (W) fractions were analyzed by immunoblotting with specific antibodies against GspA. High-molecular-mass (HMM) and low-molecular-mass (LMM) species of GspA, GspA monomer (M), and molecular mass markers are indicated. (C and D) *A. oris* cells were immobilized on nickel grids and stained with 1% uranyl acetate prior to viewing with an electron microscope. Bar, 0.5 µm.

As previously reported (15), *A. oris srtA* is an essential gene, and a screen to identify suppressors of the *srtA* lethal phenotype identified an LCP homolog (ana_1292), here named *lcpA*, which is located immediately downstream of *gspA* (Fig. 1A), another suppressor of *srtA* lethality (15). In addition to LcpA, *A. oris* MG1 encodes three additional proteins with LCP domains (see Fig. S1 in the supplemental material). ana_0299, designated *lcpB*, is adjacent to two conserved genes (Fig. 1A) coding for a UDP-*N*-acetyl-d-mannosaminuronic acid dehydrogenase (ana_0300) and a homolog of glycosyl/glycerophosphate transferase TagF, which has previously been implicated in the wall teichoic acid (WTA) synthesis of Staphylococcus epidermidis (17). ana_1577 (*lcpC*) and ana_1578 (*lcpD*) appear to reside in the same transcriptional unit (Fig. 1A). Because LcpA has been linked to GspA glycosylation (15), we examined if genetic disruption of LcpB, LcpC, and LcpD affects this process, although all three were not identified from the original suppressor screen. We obtained mutants in *lcpB* and *lcpD*, but we were unable to generate a deletion mutant of the *lcpC* gene after several attempts, suggesting *lcpC* may be an essential gene. A triple mutant (*lcp*Δ*3*), devoid of *lcpA*, *lcpB*, and *lcpD*, was also attained.

10.1128/mBio.01580-18.1 FIG S1 Sequence alignment of LCP proteins. Multiple sequence alignment of the open reading frames of *A. oris* LcpA (WP_065362445), LcpB (WP_065361955), LcpC (WP_029316527), and LcpD (BAV84788); Bacillus subtilis TagT (WP_032722634); and Streptococcus pneumoniae CpsA2 (WP_000091085) was performed by CLUSTALW (45), and the results were plotted using BoxShade (v3.21), with the fraction of sequences for shading set at 0.5. Download FIG S1, PDF file, 0.04 MB. Copyright © 2019 Siegel et al. 2019 Siegel et al. This content is distributed under the terms of the Creative Commons Attribution 4.0 International license.

To analyze LcpA-mediated glycosylation, cell cultures of *A. oris* MG1 and its derivatives were grown to mid-log phase, normalized by optical density, and subjected to cell fractionation, as previously described (15). Protein samples from the culture medium (S) and cell wall (W) fractions were analyzed by Western blotting with a specific antibody against GspA (α-GspA). As reported before (15), the MG1 strain (WT) produced a high-molecular-mass species of GspA with glycan polymers, i.e., GspA^HMM^, detected mostly in the cell wall fractions (Fig. 1B, lanes WT). Deletion of *lcpA* abrogated formation of GspA^HMM^, resulting in accumulation of low-molecular-mass species of GspA, termed GspA^LMM^, migrating around the 37-kDa marker, although the GspA monomer (GspA^M^; arrowhead) migrated at the 25-kDa marker (Fig. 1B, lanes Δ*lcpA*). The Δ*lcpB* and Δ*lcpD* single mutant strains displayed no significant defects in formation of GspA^HMM^ (Fig. 1B, lanes Δ*lcpB* and Δ*lcpD*), whereas the *lcp*Δ*3* triple mutant failed to produce GspA^HMM^, phenocopying the *lcpA* mutant; this defect was rescued by ectopic expression of *lcpA* in the *lcp*Δ*3* mutant (Fig. 1B, last 4 lanes). To determine if deletion of *lcpA* affects cell morphology and pilus assembly, the parental and *lcpA* mutant strains were examined by electron microscopy, whereby bacterial cells, immobilized on carbon-coated nickel grids, were stained with 1% uranyl acetate prior to viewing with an electron microscope. As shown in Fig. 1C and D, the two strains displayed similar cell morphology and pilus assembly phenotypes. Altogether, the results support that LcpA is necessary and sufficient to produce GspA^HMM^ and suggest that GspA^LMM^ might represent an intermediate form of the glycoprotein GspA^HMM^.

### X-ray structure of LcpA from Actinomyces oris.

**FIG 2:**
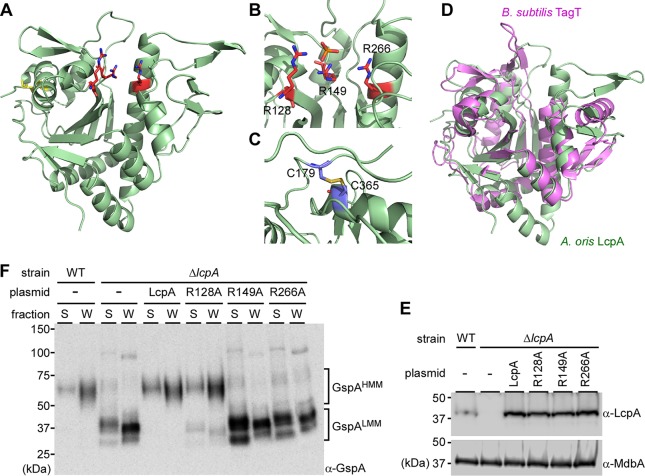
Crystal structure of *A. oris* LcpA and structural requirements for glycosylation activity. (A) The structure of the extracellular LCP domain (eLcpA, residues 78 to 370) was determined to 2.5-Å resolution. The proposed catalytic Arg residues are shown as sticks and colored red, and the cysteine residues participating in the disulfide bond are shown in yellow. (B) Presented is a detailed view of the LcpA active site, with the conserved catalytic arginine residues (R128, R149, and R266) shown in red. (C) Shown is a closeup view of the disulfide bond that links the C terminus via C365 to the second α-helix via C179 present in the LCP extracellular domain. (D) The LcpA structure (light green) is superimposed with the Bacillus subtilis YwtF (TagT) (PDB 4DE9) (magenta). (E) Protein samples of indicated strains were prepared as described in Fig. 1B and analyzed by immunoblotting with anti-GspA. (F) Protein samples from the membrane fractions in panel E were immunoblotted with antibodies against LcpA. A membrane protein, MdbA (20), was used as a control. Molecular mass markers in kDa are shown.

To obtain insight into how LcpA glycosylates GspA, we first determined the molecular structure of the LcpA enzyme. An inspection of its primary sequence reveals a tripartite structure: (i) residues 1 to 54 presumably reside in the cytoplasm and are predicated to adopt helical secondary structure, (ii) residues 55 to 77 are nonpolar and likely form a single transmembrane helix (TM), and (iii) residues 78 to 370 presumably reside on the extracellular surface and share primary sequence homology to LCP-type enzymes (Pfam family PF03816). The structure of the extracellular LCP domain (eLcpA, residues 78 to 370 [Fig. S2]) was solved at 2.5-Å resolution. Electron density was observed for residues 79 to 106 and 126 to 368, which form a single domain that adopts an α-β-α architecture. A seven‐stranded antiparallel β‐sheet forms the core of the protein with a total of eight α‐helices flanking the β-sheet on both of its faces, forming a hydrophobic tunnel (Fig. 2A; Table 1). The tunnel is ∼23 Å in length and is lined by residues located on the central β-sheet and helices H5, H6, and H7. The tunnel varies in width from ∼6 to 14 Å and is widest in the middle of the core of the protein. The surface of the tunnel contains many nonpolar residues consistent with it interacting with lipid substrates. Interestingly, during refinement additional electron density was observed near the exit point of the tunnel, defined by helices H6 and H7, indicating that a ligand was bound. However, it was not possible to conclusively determine the identity of this ligand using MALDI-TOF mass spectrometry and modeling the ligand as phosphate-isoprenoid molecule or other membrane-associated lipid yielded poor refinement statistics. The best match to the data was obtained by modeling the ligand as a PEG 4000 molecule that was used as a precipitant during crystallization. This ligand is bound with 50% occupancy and defines the exit point for the tunnel distal to the active site. The presence of a hydrophobic tunnel leading into the active site suggests that LcpA could bind a lipid-linked glycan donor substrate, similarly to other members of the LCP superfamily (7).

**TABLE 1:** Crystallographic statistics of *A. oris* LcpA

Statistic	eLcpA	Se^−^ eLcpA
Space group	P2_1_2_1_2_1_	P2_1_2_1_2_1_
Cell dimensions		
a, b, c (Å)	40.85, 69.07, 82.01	40.92, 69.19, 81.95
a, b, g (°)	90.00, 90.00, 90.00	90.00, 90.00, 90.00
Wavelength	0.97900	Peak, 0.97900; remote, 0.97170; inflection, 0.97930
Resolution (Å)	2.51–52.83	2.67–52
*R*_merge_	0.051	
*I*/*s*(*I*)	2.06	
CC_1/2_	0.998	
Completeness (%)	98.90	
Redundancy	92.9	
Refinement		
Resolution (Å)	2.51	
No. of reflections	52,941	66,067
*R*_work_/*R*_free_	0.221/0.272	

10.1128/mBio.01580-18.2 FIG S2 Purification of recombinant LcpA proteins. The LcpA soluble domain (eLcpA) (A) and mutant derivatives R149A (B) and C179A/C365A (Δs-s) (C) were cloned in the pMCSG7 vector and expressed and purified from E. coli SHuffle (C3209) cells. Protein samples were analyzed by SDS-PAGE and stained with Coomassie brilliant blue R-250. The lane numbers represent the following fractions: total protein (1), flowthrough (2), wash (3), elution (4), and postdesalt void (5). Download FIG S2, PDF file, 0.5 MB. Copyright © 2019 Siegel et al. 2019 Siegel et al. This content is distributed under the terms of the Creative Commons Attribution 4.0 International license.

Members of the LCP superfamily contain conserved arginine residues (Fig. S1), which are thought to mediate a phosphotransfer reaction that attaches glycopolymers to acceptors (7, 18, 19). In *A. oris* LcpA, R128, R149, and R266 are conserved residues (Fig. S1), which cluster together within a surface-exposed pocket (Fig. 2A and B, shown in red). One surface of this exposed active site is formed by residues in strand β3 and helix H1, while the top and side of the pocket are formed by helix H4 and H5, respectively, packing against the core β-sheet. R128 and R149 in the pocket are positioned toward the surface and located in strands β3 and β4/β5 loop, respectively. Helix H5 spans the length of the protein and contains the third conserved active arginine (R266), which is located closer to the body of the enzyme where the pocket narrows. Electron density is observed between the guanidino side chains of R128 and R149 and the modeled phosphate atom. The hydrophobic tunnel leads from this conserved site to the opposite face of the protein structure.

Intriguingly, unlike other LCP enzymes, LcpA contains a disulfide bond, formed between residues C179 and C365, linking the C terminus to α-helix H2 (Fig. 2C). This disulfide is presumably stabilizing, since it persists despite the presence of a reducing agent in the protein buffer used in the final purification step and the cysteine residues are conserved in other LCP homologs present in *Actinobacteria* (Fig. S3).

10.1128/mBio.01580-18.3 FIG S3 Phylogenetic analysis of LCP proteins. The tree was rooted with the HD domain-containing protein from Thermotoga maritima (WP_004082198) and constructed by using the mega6 program. Numbers at nodes represent percent levels of bootstrap support based on the unweighted pair group method with arithmetic mean of 1,000 resampled data sets. Boldface indicates actinobacterial LCP proteins that contain cysteine residues. Download FIG S3, PDF file, 0.07 MB. Copyright © 2019 Siegel et al. 2019 Siegel et al. This content is distributed under the terms of the Creative Commons Attribution 4.0 International license.

The structure of eLcpA is similar to that of previously reported LCP enzymes that attach polymers to the cell wall and is most closely related to the YwtF (TagT) enzyme from Bacillus subtilis based on a DALI analysis (Z-score of 21.8 with PDB 4DE9 [18]); the backbone atoms can be superimposed with a root mean square deviation (RMSD) of 2.5 Å (Fig. 2D). The structural conservation and presence of arginine residues in the surface-exposed pocket prompted us to investigate functional similarities to TagT related to GspA glycosylation.

### The conserved arginine residues in LcpA are required for glycosylation activity.

As presented above, LcpA is required for glycosylation of GspA (Fig. 1B) and LcpA contains conserved arginine residues (R128, R149, and R266) (Fig. 2B). Conserved Arg residues have been implicated in LCP activity by interacting with the pyrophosphate of the lipid-linked glycan donor (7). To determine whether these Arg residues affect the glycosylation activity of *A. oris* LcpA, we generated alanine substitution mutants of these arginine residues using pLcpA as a template (Fig. 1B). Plasmids expressing mutant proteins were introduced into the Δ*lcpA* mutant, and expression of LcpA proteins was determined by immunoblotting membrane lysates of various strains with specific antibodies against LcpA (α-LcpA), with α-MdbA being used as a control for the membrane-bound protein MdbA (20). As expected, LcpA was detected in the parental strain and absent from the Δ*lcpA* mutant (Fig. 2E, first two lanes). Complementation of the Δ*lcpA* mutant with a multicopy plasmid enhanced LcpA expression, compared to the WT strain (Fig. 2E, lane LcpA). Mutations of the three Arg residues did not affect the expression of mutant proteins compared to ectopically expressed wild-type LcpA (Fig. 2E, last 3 lanes). We then examined GspA glycosylation by Western blotting the supernatant and cell wall fractions as described in Fig. 1B. Interestingly, the LcpA-R128A mutant was able to produce GspA^HMM^ at the level comparable to that of the WT strain and the Δ*lcpA*/LcpA rescued strain, whereas the LcpA-R149A and LcpA-R266A mutants were defective in glycosylation of GspA, matching the Δ*lcpA* mutant (Fig. 2F). Altogether, the results support that the R149 and R266 residues are essential for the glycosylation activity of LcpA.

### The disulfide bond in *A. oris* LcpA is required for protein stability.

**FIG 3:**
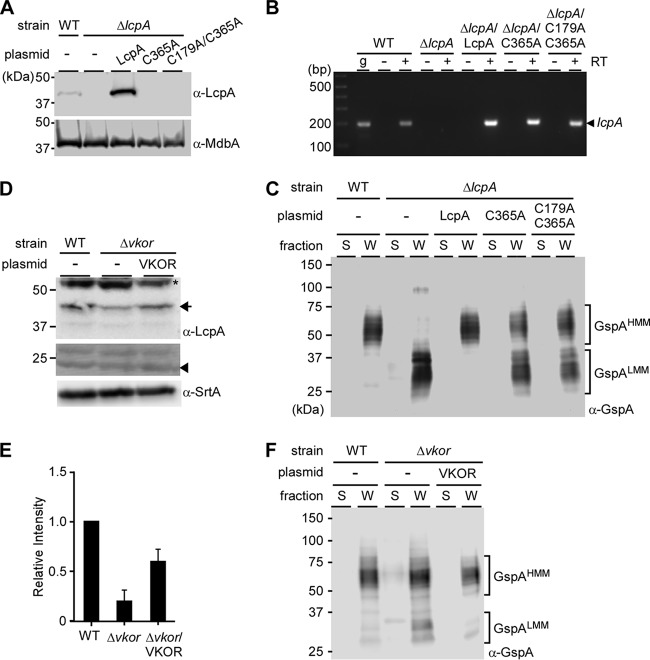
The disulfide bond C179-C365 is required for LcpA stability. (A) Immunoblotting of the membrane fractions of indicated strains was performed as described in Fig. 2F with anti-MdbA used for the control membrane protein MdbA. (B) Expression of *lcpA* in indicated strains was analyzed by RT-PCR using primers specific for a 196-bp region of *lcpA*. *A. oris* MG1 genomic DNA (gDNA) was used as controls for length and specificity. + and − indicate the presence or absence, respectively, of reverse transcriptase (RT). (C) Protein samples from the indicated strains were prepared and analyzed by immunoblotting as described in Fig. 2F. (D) Shown is a representative Western blot of protoplast fractions of the MG1 strain (WT), the Δ*vkor* mutant, and this mutant expressing VKOR from a plasmid. LcpA is marked with an arrow, whereas a nonspecific band is shown as an asterisk; an arrowhead is used for a loading control band from the immunoblotted membrane stained with Coomassie blue. The membrane-bound protein SrtA serves as a control. (E) Relative expression of LcpA was determined by comparing the relative intensities of the LcpA bands in panel D, which were normalized against the loading control band. The relative intensity of the wild-type LcpA bands was set to 1. Error bars represent the standard deviation (SD) for 4 independent replicates. (F) The culture medium and cell wall fractions of the indicated strains were analyzed by immunoblotting as described in panel C.

*A. oris* LcpA has a stable disulfide bond (Fig. 2C) that appears to be a conserved feature in actinobacterial Lcp proteins (Fig. S1). To determine the role of the disulfide bond in LcpA stability and glycosylation activity, we generated alanine substitution mutants of either one (C365) or both Cys (C179 and C365) residues. Membrane fractions of the parental and mutant strains were immunoblotted with α-LcpA as described in Fig. 2E. Consistently, enhanced signal of LcpA was observed in the strain expressing LcpA from a plasmid, compared to the parental strain (Fig. 3A, first 3 lanes). However, no LcpA signal was detected in the membrane of strains expressing LcpA with the C365A or C179A/C365A mutation (Fig. 3A, last 2 lanes), suggesting that the disulfide bond formed by C179 and C365 is required for membrane expression of LcpA. To further assess if this disulfide bond is required for LcpA stability, recombinant LcpA proteins (wild type, C179A/C365A, and R149A) were used in a thermal stability assay that is based on the ability of SYPRO orange to bind to hydrophobic surfaces of proteins exposed by the unfolding process; the unfolding temperatures or melting temperatures (*T_m_*) are then determined (21). As shown in Fig. S4, wild-type and R149A proteins exhibited similar *T_m_*, whereas a melting temperature for the C179A/C365A mutant could not be accurately calculated, likely due to low initial protein stability. It is noteworthy that LcpA contains a large hydrophobic cleft (Fig. 2A), the exposure or stability of which might be severely affected by the absence of the disulfide bond C^179^-C^365^.

10.1128/mBio.01580-18.4 FIG S4 Requirement of disulfide bond formation for LcpA thermal stability. Recombinant LcpA proteins—wild-type and its R149A and C179A/C365A mutants—were used in a Thermofluor assay in a 96-well PCR plate. Each reaction, with the mixture containing 45 μl of protein solution (5 mM) and 5 μl of 200× SYPRO orange solution, was performed in a Bio-Rad CFX real-time PCR system. The melting temperature (*T_m_*) was obtained from three independent experiments performed in triplicate. Error bars represent standard deviation. Download FIG S4, PDF file, 0.03 MB. Copyright © 2019 Siegel et al. 2019 Siegel et al. This content is distributed under the terms of the Creative Commons Attribution 4.0 International license.

To ensure the expression defect shown in Fig. 3 above was not due to the deficiency of *lcpA* transcripts, we collected mRNA in these strains and used reverse transcription-PCR (RT-PCR) to amplify a 196-bp region specific to the *lcpA* gene. In the WT strain, the *lcpA* transcript was detected only when reverse transcriptase (RT) was added, with *lcpA* amplified from genomic DNA (gDNA) used as a control for the length and specificity of the amplicon (Fig. 3B, lanes WT). As expected, no *lcpA* transcript was detected in the *lcpA* mutant (Fig. 3B, lanes Δ*lcpA*), while the transcript levels of *lcpA* expressed from these recombinant plasmids were comparable to and significantly higher than the *lcpA* level in the WT strain (Fig. 3B, remaining lanes). Altogether, the results suggest that the defect of LcpA membrane expression above is not due to lack of gene expression but rather due to protein instability due to the absence of the disulfide bond.

We next examined if mutations of these cysteine residues affect LcpA glycosylation activity by immunoblotting the culture medium and cell wall fractions of the same set of strains, according to the procedure described in Fig. 2F. Surprisingly, strains expressing LcpA with C365A or C179A/C365A mutation produced GspA^HMM^, albeit less abundantly than the WT and rescued strains, with accumulation of the intermediate GspA^LMM^ unlike the abovementioned strains (Fig. 3C). The data support that the disulfide bond is necessary for full activity of LcpA.

We previously reported that disulfide bond formation in *A. oris* requires the activity of a membrane-bound thiol-disulfide oxidoreductase named MdbA (20), and reactivation of MdbA involves another oxidoreductase called VKOR (22, 23). Because *mdbA* is an essential gene, whereas a mutant devoid of *vkor* is viable although it exhibits severe defects in oxidative protein folding (20), we examined if LcpA stability is affected in the *vkor* mutant. To test this possibility, the parent, its isogenic Δ*vkor* mutant, and rescued strains were subjected to cell fractionation. To determine if deletion of *vkor* affects LcpA expression, protoplast fractions were analyzed by Western blotting with anti-LcpA; protein levels were quantified by densitometry from four independent experiments with loading controls from the same blots stained with Coomassie blue. Compared to the WT and rescued strains, the Δ*vkor* mutant produced significantly less LcpA (Fig. 3D and E). As a control, the protein level of the housekeeping sortase SrtA remained the same in three strains (Fig. 3D). When the culture medium and cell wall fractions were immunoblotted with anti-GspA, no significant defects of GspA glycosylation were observed, evident by formation of GspA^HMM^ detected in the Δ*vkor* mutant, and the GspA^LMM^ species accumulated in this strain compared to the WT and complementing strains (Fig. 3F). Altogether, the results support that disulfide bond formation is critical for LcpA stability, and this oxidative protein folding is mediated by the major oxidoreductase machine MdbA/VKOR as previously reported (20).

### LcpA exhibits pyrophosphatase activity *in vitro*.

**FIG 4:**
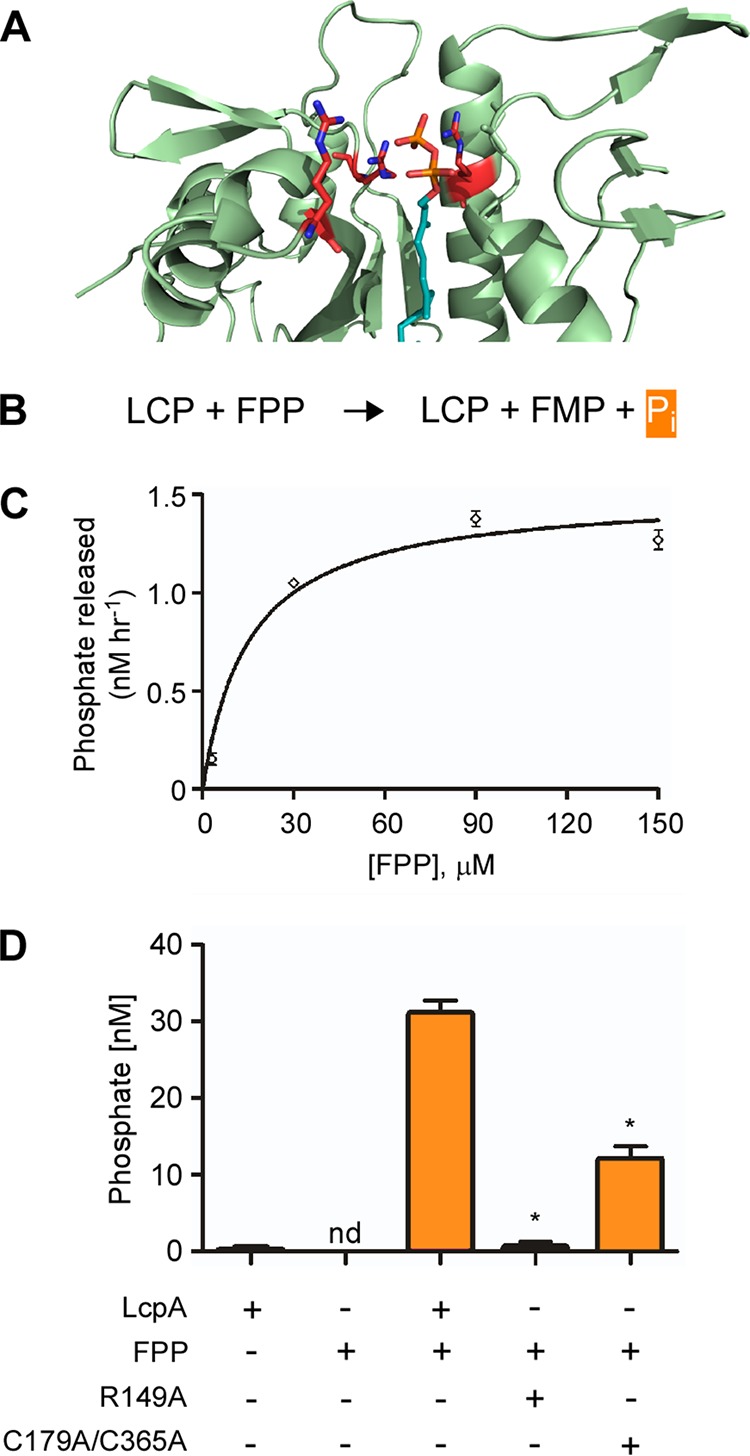
LcpA exhibits pyrophosphatase activity. (A) Octaprenyl-pyrophosphate (Opr-PP) bound to *A. oris* LcpA was modeled, with the prenol chain shown in turquoise and the pyrophosphate in orange. Potential interactions of Arg residues (red) with the pyrophosphate head group are presented. (B) Presented is the hydrolysis reaction of farnesyl pyrophosphate (FPP) by LCP enzymes resulting in formation of farnesyl monophosphate (FMP) and inorganic phosphate (P_i_, orange). (C) Recombinant LcpA at 3 μM was incubated with increasing concentrations of FPP for 24 h at 30°C. Released P_i_ was detected by a fluorescent method and quantified from three biological replicates; the *V*_max_ and *K_m_* values were calculated using the Michalis-Menten equation in Prism GraphPad, and error bars represent standard error of the mean (SEM), fitted by nonlinear regression. (D) Pyrophosphatase activity at saturating substrate concentrations (1:30) of recombinant LcpA and mutant derivatives, LcpA^R149A^ and LcpA^C179A-C365A^, was determined as described in panel C, with LcpA alone and FPP included as controls. The results were derived from three independent experiments performed in triplicates, with phosphate standards performed in parallel. Error bars represent SEM, and statistical analysis with a one-tailed Mann-Whitney-Wilcoxon test was performed using Prism GraphPad. The asterisk indicates *P* values of 0.0383 and 0.0500 for reactions with R149A and C179A-C365A enzymes, respectively; nd, not detected.

It appears that LCP enzymes studied to date possess pyrophosphatase activity, i.e., hydrolysis of diphosphate phosphoanhydride bonds. This is evident by initial characterization of the LCP enzyme TagT from B. subtilis showing that the enzyme exhibits pyrophosphatase activity *in vitro*, and this has also been demonstrated for LCP proteins of Mycobacterium tuberculosis and C. glutamicum (7, 9, 10). To examine if this is the case in *A. oris*, we utilized an *in vitro* assay with a diphosphate mimetic substrate, farnesyl pyrophosphate (FPP), and eLcpA and its mutant derivatives expressed in and purified from Escherichia coli (Fig. S2). We modeled an octaprenyl‐pyrophosphate (Opr-PP) molecule into the LcpA hydrophobic pocket by using the structure of the TagT enzyme bound to all *cis-*Opr‐PPs (PDB 4DE9) to model the structure of the Opr-PP:eLcpA complex; this was achieved by superimposing the protein coordinates, as well as the coordinates of the phosphate proximal to the glycan strand in the structure of TagT and the active site phosphate atom present in the structure of eLcpA. Subsequently, the model was created using the electron density of the modeled phosphate ion to place the phosphate head groups of Opr-PP and the electron density used to model PEG 4000 to model the lipid component of the Opr-PP polyprenol (Fig. 4A). Pyrophosphatase activity of LcpA proteins was determined by quantitatively measuring inorganic phosphate (P_i_) release from FPP (Fig. 4B). Significantly, it was found that eLcpA was able to hydrolyze FPP, exhibiting a *V*_max_ of 1.51 ± 0.08 nM h^−1^ and *K_m_* of 15.2 ± 3.7 µM (Fig. 4C). The saturating substrate concentration occurred at an enzyme-to-substrate ratio of approximately 1:30 (Fig. 4C).

We then examined if mutations of the catalytic residue R149 and disulfide bond C179/C365 affect the pyrophosphatase activity of LCP using the above-described assay with the saturating substrate concentration. As expected, the LcpA enzyme and FPP contained little to no background P_i_ (Fig. 4D, first 2 columns). Compared to the wild-type eLcpA enzyme, alanine substitution of R149 in eLcpA abrogated the enzymatic pyrophosphatase activity (Fig. 4D, compare column 4 to column 3), further confirming the essential role of this catalytic residue. Consistent with the *in vivo* results above, the eLcpA protein lacking the disulfide bond C179-C365 exhibited significantly reduced pyrophosphatase activity, approximately 3-fold less than the wild type (Fig. 4D, last column). Altogether, the results indicate that LcpA possesses pyrophosphatase activity and that the disulfide bond C179-C365 plays in important role in maintaining the full activity of LcpA.

### *A. oris* LcpA catalyzes phosphotransfer.

**FIG 5:**
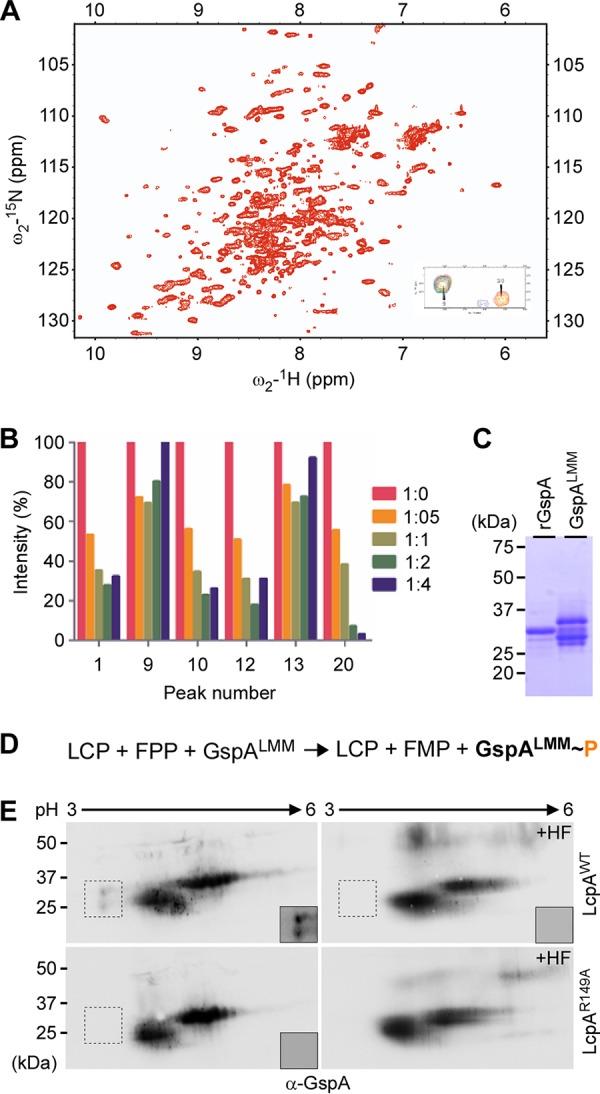
eLcpA interacts with rGspA in solution and catalyzes phosphotransfer. (A) Presented is the full ^1^H-^15^N HSQC of 250 µM ^15^N-recombinant LcpA with inset showing representative data of overlaid ^1^H-^15^N HSQC titration spectra displaying two isolated peaks, with a high signal-to-noise ratio. Red spectra represent 1:0 molar equivalents of ^15^N-eLcpA to ^14^N-rGspA. Orange, light green, dark green, and violet represent 1:0.5, 1:1, 1:2, and 1:4 spectra, respectively. Peak 9 is an example which does not exhibit a dose-dependent decrease in peak height upon adding rGspA, and peak 20 is shown as an example which does exhibit drastic effects on peak height. (B) Normalized plot of peak intensity of selected residues from titration experiment with high signal-to-noise ratio. Intensity data were normalized to 1:0 titration peak intensities. (C) Recombinant GspA (rGspA) and GspA^LMM^ were purified by affinity chromatography from E. coli and *A. oris* lysates, respectively, and analyzed by SDS-PAGE and Coomassie blue staining. (D) Presented is a simplified schematic for *in vitro* phosphotransfer. (E) The phosphotransfer reaction mixture contained 12 µM GspA^LMM^, 4 µM LcpA (WT or R149A), and 50 µM FPP in 20 mM Tris-HCl (pH 8.0). After a 72-h incubation at 30°C, protein samples were treated with hydrofluoric acid (HF) or mock treated prior to 2-D electrophoresis, followed by immunoblotting with anti-GspA antibodies. Insets with increased contrast were shown for boxed regions.

To further define the mechanism of surface protein glycosylation by LcpA, we investigated its interactions with its GspA substrate using solution NMR spectroscopy, which can detect transiently interacting proteins. ^1^H-^15^N heteronuclear single quantum coherence (HSQC) titration studies were performed with ^15^N-isotopically enriched eLcpA and ^14^N-rGspA, a truncation of GspA lacking its predicted N-terminal signal peptide and C-terminal transmembrane region. A series of ^1^H-^15^N HSQC NMR spectra of ^15^N-eLcpA with various amounts of the ^14^N-rGspA was acquired. The various spectra of the ^15^N-eLcpA (up to 1:4 ratio of ^15^N-rLcpA to ^14^N-rGspA) titrations were partially resolved, enabling line-shape, specifically peak-height, analysis (Fig. 5A). Spectra of ^15^N-eLcpA and ^14^N-rGspA at a 1:8 ratio, respectively, are completely broadened, due to either sample dilution or, more likely, spin-diffusion caused by complex formation. Unfortunately, due to the low quality of the spectra, site-specific interactions or chemical-exchange equilibria could not be estimated. However, analysis of 43 resolved peaks revealed that 4 of these peaks with high signal-to-noise ratios (approximately 20-fold over background), i.e., peaks 1, 10, 12, and 20, did exhibit a dose-dependent reduction in peak-height during the titration experiment (Fig. 5B). This suggests that eLcpA and rGspA interact weakly *in vitro*. Further refinement of this interaction will help define the LcpA-mediated mechanism of glycopolymer transfer, and these data support further studies of these interactions.

The structural evidence, pyrophosphatase activity, *in vivo* glycosylation, and enzyme-substrate interaction above are consistent with the phosphotransfer activity of LCP enzymes that have previously been shown to mediate WTA synthesis (7); WTA is linked to the *N*-acetylmuramic acid component of the cell wall via a phosphodiester linkage (1, 24). To examine if *A. oris* LcpA possesses phosphotransfer activity, we employed an *in vitro* phosphotransfer assay, in which the recombinant enzyme rLcpA was mixed with FPP and GspA proteins (Fig. S1). After 72 h of incubation at 30°C, protein samples were analyzed by 2-D gel electrophoresis, followed by immunoblotting with α-GspA. Because deletion of *lcpA* results in the accumulation of several GspA^LMM^ species (Fig. 5C), we surmised that the GspA^LMM^ proteins are substrates of LcpA that could explain their weak interaction observed by NMR as shown above. To facilitate purification of GspA^LMM^ in *A. oris*, we engineered a GspA protein with its CWSS replaced by a His tag, and the recombinant protein was expressed in the Δ*lcpA* mutant; GspA^LMM^ proteins were purified from the culture medium by affinity chromatography. Compared to the recombinant protein rGspA, which was used in Fig. 5A, the GspA^LMM^ proteins migrated between the 25-kDa and 37-kDa markers (Fig. 5C). The identity of these GspA proteins was also confirmed by mass spectrometry. If the GspA^LMM^ proteins are substrates of LcpA, addition of LcpA and FPP leads to phosphate modifications of GspA (Fig. 5D), hence increasing acidity due to the negatively charged phosphate group. As shown in Fig. 5E, in the presence of the wild-type LcpA enzyme, two new spots migrating between the 25-kDa and 37-kDa markers and toward the acidic pI were detected, compared to samples treated with the inactive enzyme LcpA^R149A^.

To test if phosphate modification of GspA^LMM^ occurs via a phosphodiester bond, the duplicate samples, i.e., LcpA + FPP + GspA^LMM^, were treated with hydrofluoric acid (HF) prior to 2-D gel electrophoresis and immunoblotting; HF hydrolyzes phosphodiester bonds as previously demonstrated in S. aureus with an LCP enzyme but leaves glycosidic and peptide bonds intact (25). Indeed, HF treatment resulted in abrogation of phosphate modification (Fig. 5E, HF panels). Altogether, the results support that LcpA is a phosphotransferase and that GspA^LMM^ is a bona fide substrate for LcpA-catalyzed glycosylation.

## DISCUSSION

Members of the LCP protein family studied to date have been shown to attach glycopolymers to peptidoglycan (5, 6), with many demonstrated to possess pyrophosphatase and phosphotransferase activities (7, 9, 10, 26, 27). LCP enzymes are characterized as the terminal enzyme which catalyzes the linkage of glycopolymers to the muramic acid component of the peptidoglycan via a phosphodiester bond from a prenyl pyrophosphate glycan donor (8, 28). None of these enzymes, however, have been shown to mediate glycosylation of cell wall-anchored proteins. We present here the first experimental evidence that *A. oris* LcpA—capable of catalyzing hydrolysis of diphosphate bonds and phosphotransfer—glycosylates the cell wall-anchored protein GspA prior to its attachment to peptidoglycan, a subsequent process that is facilitated by the housekeeping sortase SrtA (15).

We report here that crystallization studies authenticate LcpA as a member of the LCP protein family, revealing that it is structurally related to the B. subtilis TagT enzyme that mediates the linkage of WTAs to peptidoglycan (18). The two enzymes have similar hydrophobic tunnels that are capped with Arg residues (R149 and R266 in *A. oris* LcpA), a conserved feature of LCP enzymes that is necessary for interaction with glycan donor substrates. Our mutagenesis results indicate that Arg149 and Arg266 have important roles in catalysis (Fig. 2F). This is consistent with very recently published mechanistic studies of B. subtilis TagT reported by Schaefer et al. (11). The residue analogous to Arg149 in *A. oris* LcpA (Arg118 in TagT) is important for catalysis and is proposed to function as a general base that deprotonates the C-6-hydroxyl of MurNac in the WTA substrate. In LcpA, Arg149 may therefore be required to deprotonate a hydroxyl group within GspA to which the polymer is attached, consistent with our observation that R149A mutation also disrupts the *in vitro* pyrophosphatase activity of LcpA. The analog of Arg266 in LcpA (Arg227 in TagT) is also important for TagT activity and may stabilize the pyrophosphoryl-oxygen within the WTA substrate. In LcpA, a similar role in catalysis can be envisioned in which it stabilizes the pyrophosphate unit within the lipid-linked glycopolymer substrate. Finally, our finding that Arg128 is dispensable for GspA modification is also consistent with their results, as the analogous residue in TagT (Arg95) is distal to the WTA substrate in the crystal structure of the TagT-substrate complex.

Unlike B. subtilis TagT, *A. oris* LcpA does not appear to attach glycopolymers to peptidoglycan, as a GspA mutant lacking the CWSS still contains glycans (15); our current data in Fig. 5 with LcpA-mediated phosphorylation of recombinant GspA^LMM^, a molecule lacking the CWSS, also support this notion. This raises an intriguing question as to where glycopolymers are attached to in GspA. While the biochemical nature of the glycans and glycosylation sites remains to be elucidated, the results presented in our previous publication (15) and Fig. 5 suggest that an intermediate form of GspA, GspA^LMM^, may serve as a substrate of LcpA. It is interesting that *A. oris* harbors four LCP homologs but only *LcpA* is involved in GspA glycosylation (Fig. 1). Because a conditional deletion mutant is not available, this does not exclude the possibility that LcpC may modify GspA, leading to formation of GspA^LMM^. Future experiments will address this issue.

Intriguingly, the presence of a hydrophobic tunnel in the LcpA and TagT structures as mentioned above suggests that the enzymes use a pyrophosphate-lipid-linked glycan donor; a model of the Opr-PP:eLcpA complex was subsequently generated (Fig. 4A). This model suggests that eLcpA catalyzes a phosphotransfer reaction in which the pyrophosphate linkage joining the lipid to the sugar molecule is broken, presumably as a result of nucleophilic attack by an oxygen or nitrogen atom present on an amino acid side chain within the GspA protein. As a result, the proximal phosphate and glycan are transferred to GspA. Based on studies of TagT, R149 may function as a base that deprotonates a nucleophile originating from GspA, whereas R266 may favorably interact with the trigonal bipyramidal intermediate that likely forms during catalysis. The process is thermodynamically favorable, as breakage of the phosphoanhydride linkage in the substrate releases more free energy than is required to attach a sugar molecule to the protein (the Gibbs standard free energy for phosphoanhydride breakage in the substrate is ∼−7.3 kcal/mol, whereas only ∼3.3 kcal/mol is required to form the phosphodiester bond that joins the sugar to the protein). The complexity of the glycans has prohibited our ability to determine the exact identity of our glycan species, although this is a subject of current work.

In contrast to other LCP proteins studied to date, *A. oris* LcpA possesses a distinct feature, which appears to be commonly present in the actinobacterial LCP enzymes, i.e., a disulfide bond. Given that disulfide bond formation is critical for oxidative folding of exported proteins in *Actinobacteria* (29), a process that is catalyzed by a pair of thiol-disulfide oxidoreductase enzymes, MdbA/VKOR, in *A. oris* (20), we hypothesized that the disulfide bond formed between C179 and C365 is essential for posttranslocational folding of LcpA. This is evidenced by the fact that mutations that abrogate the disulfide bond C179-C365 severely affect membrane expression of LcpA, whereas deletion of VKOR significantly reduces LcpA membrane expression (Fig. 3).

**FIG 6:**
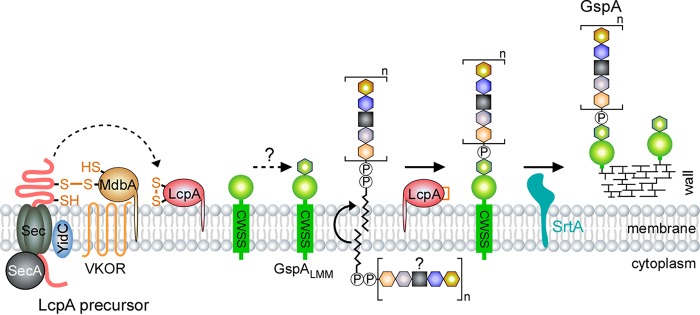
Proposed model of LcpA-mediated glycosylation of the cell wall-anchored protein GspA*. A. oris* LcpA is proposed to catalyze the linkage of unknown glycopolymers to GspA, which is then anchored to the bacterial cell wall by the housekeeping sortase SrtA. The oxidoreductase enzymes MdbA/VKOR are thought to catalyze oxidative folding of LcpA (see the text for details).

Altogether, we propose a model that as the LcpA precursor emerges from the Sec machine, it is folded by the MdbA/VKOR enzymes and inserted into the membrane by the membrane protein insertase YidC. Separately, the membrane-bound GspA is also transported by the Sec and further modified by an unknown mechanism, resulting in an intermediate form named GspA^LMM^. LcpA catalyzes the attachment of an unknown glycan chain to GspA^LMM^, which is then anchored to the bacterial peptidoglycan by the housekeeping sortase SrtA (Fig. 6). Given the conservation of LCP and GspA proteins, it is likely that this glycosylation pathway is conserved in *Actinobacteria*. Nonetheless, whether *A. oris* LcpA is capable of glycosylating peptidoglycan or not remains to be investigated.

## MATERIALS AND METHODS

### Bacterial strains, plasmids, and media.

*Actinomyces* strains were grown in heart infusion broth (HIB) and on heart infusion agar (HIA) at 37°C with 5% CO_2_. E. coli strains were grown in Luria-Bertani broth or Luria-Bertani agar at 37°C. If needed, 50 µg ml^−1^ kanamycin (Kan) or 100 µg ml^−1^ ampicillin (Amp) was added to bacterial cultures. E. coli strain DH5α was used for cloning experiments, and E. coli SHuffle (C3209) was used for protein expression (30). A complete list of strains and plasmids can be found in Tables S1 and S2 in the supplemental material.

10.1128/mBio.01580-18.5 TABLE S1 Bacterial strains and plasmids used in this study. Download Table S1, PDF file, 0.05 MB. Copyright © 2019 Siegel et al. 2019 Siegel et al. This content is distributed under the terms of the Creative Commons Attribution 4.0 International license.

10.1128/mBio.01580-18.6 TABLE S2 Primers used in this study. Download Table S2, PDF file, 0.06 MB. Copyright © 2019 Siegel et al. 2019 Siegel et al. This content is distributed under the terms of the Creative Commons Attribution 4.0 International license.

### Plasmid construction.

A detailed list of plasmids and primers can be found in Table S2. (i) For prLcpA-SUMO, DNA encoding soluble LcpA, residues 78 to 370, from the MG1 strain of *A. oris* was cloned into the pE-SUMO vector (LifeSensors) using the Gibson assembly method (New England Biolabs) (31). (ii) For prLcpA,: *A. oris* LcpA (ana_1292) sequence containing residues 78 and 370 (lacking the transmembrane region) was amplified from the *A. oris* MG1 genomic DNA template with the primers rLcpA-F and LCP^ΔTM^-R (Table S2), containing adapter sequences for subsequent ligation-independent cloning (LIC) into pMCSG7 (32). (iii) For prGspA, the *A. oris* GspA (ana_1291) sequence containing only residues 31 and 256 (lacking signal peptide and cell wall sorting signal) was amplified from the *A. oris* MG1 genomic DNA template with the primers rGspA-F and rGspA-R (Table S2), containing adapter sequences for subsequent ligation-independent cloning (LIC) into pMCSG7. The resultant amplicons from steps i and ii were treated with LIC-qualified T4 DNA polymerase (New England Biolabs) and dCTP. In parallel, the SspI-digested vector was treated with T4 DNA polymerase and dGTP. The insert and vector fragments were ligated in a step-down annealing reaction. The vector was transformed into chemically competent E. coli DH5α. (iv) For pLcpA, the *A. oris* LcpA (ana_1292) complement strain was cloned by amplifying the native promoter, 152 bp upstream of the start codon until the native stop codon, using primers pLCP-F and pLCP-R, engineered with a BamHI and EcoRI cut site, respectively, and using *A. oris* MG1 genomic DNA as a template. The resultant amplicon was gel purified. Simultaneous restriction digestion with BamHI-HF and EcoRI-HF (NEB) of the amplicon and parental plasmid (pCWu10) was performed, and the products were again gel purified. The restricted products were ligated with T4 DNA ligase at 16°C for 18 h. The ligation mixture was transformed into chemically competent E. coli DH5α, and colony PCR was used to identify plasmids with the correct insert. The plasmid was introduced to *A. oris* Δ*lcpA* cells via electroporation (33).

### Site-directed mutagenesis.

According to a previously published procedure (34), alanine substitutions were generated using inverse PCR and intramolecular ligation with appropriate primers (Table S2), the 5′ ends of which contain mutations generated by PCR amplification. The PCR products were purified by gel extraction, and the 5′ end was phosphorylated with T4 polynucleotide kinase (New England Biolabs) to permit recircularization of the linear amplicon during the ligation step. The generated plasmid was transformed into DH5α, and the *lcpA* gene was sequenced to ensure the mutation was present and in frame prior to transformation into *A. oris*.

### Protein purification.

(i) For eLcpA-SUMO, selenomethionine-labeled eLcpA-SUMO was produced by expressing the proteins in M9 minimal medium supplemented with selenomethionine (Sigma). Briefly, the cultures are incubated at 37°C until the OD_600_ reaches 0.6 units, equilibrated to 17°C, and induced with 1 mM IPTG. Proteins were purified as a His_6_-SUMO-eLcpA fusion by immobilized-metal affinity chromatography (IMAC) using HisPure Co^2+^ resin (Thermo) per manufacturer instructions in 50 mM Tris-HCl, pH 7.5, 250 mM NaCl, and 5 mM MgCl_2._ The His_6_-SUMO tag was removed by the addition of Ulp1 protease and subsequent HisPure Co^2+^ purification. A final gel filtration purification step was carried out using a Superdex 75-pg column equilibrated in 50 mM Tris-HCl, 100 mM NaCl, 5 mM MgCl_2_, and 5 mM DTT. Purity and identity were confirmed by SDS-PAGE and MALDI-MS. (ii) eLcpA and alanine substitution mutants, generated as described above, were introduced into E. coli SHuffle (C3209) for protein purification. The strains were inoculated into 500 ml LB supplemented with 100 µg ml^−1^ Amp and grown to an OD_600_ of 0.8 at 30°C. Protein expression was induced using 0.1 mM IPTG, and the culture was transferred to 16°C overnight. Cells were pelleted by centrifugation and washed by suspension in EQ buffer (150 mM NaCl, 50 mM Tris HCl, pH 7.4). Cells were treated with 1× protease inhibitor cocktail (GenDEPOT) and lysozyme for 2 h at 37°C in EQ buffer. The treated cells were chilled and then lysed. Lysates were centrifuged to remove cell debris and nucleic acids. The remaining soluble fraction was decanted onto an equilibrated Ni-NTA column (Qiagen). The bound proteins were washed with 100 mM imidazole to remove nonspecific proteins and eluted with 500 mM imidazole in wash buffer (1× EQ and 10% glycerol). Imidazole was removed from the eluates with a desalting column (Bio-Rad), exchanged with wash buffer (Fig. S1). (iii) rGspA was purified similarly but washed with 20 mM (W1), 30 mM (W2), and 50 mM (W3) imidazole, and then elution fractions were collected in 1 ml at 100 mM (E1), 200 mM (E2 and E3), and 500 mM (E4 and E5). Fractions E1, E4, and E5 were pooled, desalted, and concentrated. (iv) GspA^LMM^ was purified from *A. oris* according to a previously published protocol (34). Briefly, *A. oris* cells expressing pGspA_Δcws_-H6 in the wild-type (MG1) background were used to generate GspA^HMM^ and those in the Δ*lcpA* background were used to generate GspA^LMM^. Cell-free supernatant from mid-log-phase cultures was collected and incubated with nickel-NTA resin overnight at 4°C. Then, the resin was decanted onto a column and washed. Proteins were eluted with 5 ml of 500 mM imidazole, desalted, and concentrated as above.

### Crystallization and diffraction data collection, processing, and structure determination.

Crystals of eLcpA were generated for structure determination with selenomethionine by concentrating eLcpA to approximately 15 mg/ml. Crystals of eLcpA were obtained using the hanging-drop vapor diffusion method by mixing protein 1:1 with 0.1 M sodium citrate, pH 5.5, 25% PEG 4000, 20% 2-propanol mother liquor. For X-ray data collection, cryoprotection was not necessary for eLcpA. Diffraction data sets were collected at the Advanced Photon Source (APS) beamline 24-1D-C equipped with a Pilatus-6M detector. All data were collected at 100 K. For multiple-wavelength anomalous diffraction (MAD) phasing, data were acquired using three independent wavelengths, with 0.5° oscillations. Selenomethionine eLcpA crystals diffracted to 2.67-Å resolution, whereas native eLcpA crystals diffracted to 2.5-Å resolution. The XDS/XSCALE package was used to index, integrate, and scale data in the P2_1_2_1_2_1_ space group (35). Phasing information was obtained using the SHELX package (36). The asymmetric unit of the crystal contained a single protein molecule, yielding a Matthews coefficient of 1.87 Å/Da and a 34.16% solvent content in the crystal. Initial refinement used the Phenix software packages; to complete refinement with all modeled ligands, we utilized BUSTER (37, 38). Model building was done using Coot (39). A bound PEG 4000 molecule was modeled with 50% occupancy. Complete refinement and structure statistics are reported in Table 1.

### Affinity purification of rabbit-raised antibodies.

Rabbit-raised antibodies against eLcpA (Cocalico Biologicals, Inc.) were subjected to affinity purification as follows. Purified eLcpA at 1.2 mg was separated by SDS-PAGE and blotted onto a PVDF membrane, which was stained with Ponceau S. Membrane strips containing LcpA were blocked with 5% skim milk in Tris-buffered saline with Tween 20 (TBST) and then treated with 5 ml of anti-LcpA antibodies overnight. Antibody-bound membranes were washed with TBST, and the antibodies were eluted with 100 mM glycine (pH 2.5) in TBST. The eluates were neutralized by addition of 2 M Tris-HCl (pH 8.5). The antibodies were dialyzed against Dulbecco’s phosphate-buffered saline (PBS) (pH 7.4) overnight.

### Cell fractionation and immunoblotting.

*A. oris* cells were grown to early log phase (0.3 to 0.4) and normalized to an OD_600_ of 0.5. The cells were subjected to fractionation as previously reported (15). Protein samples from supernatant (S), cell wall (W), cytoplasmic membrane (M), and cytoplasmic (C) fractions were analyzed with 3 to 20% SDS-PAGE gels and immunoblotted with specific antibodies (1:10,000 dilution for anti-GspA; 1:1,000, affinity-purified anti-LCP; 1:8,000, anti-SrtA). The proteins were detected by chemiluminescence using a secondary anti-rabbit antibody conjugated to HRP.

LcpA signal in different Western blots was normalized against a Coomassie blue-stained loading control band from the same blot and quantified using ImageJ, https://imagej.nih.gov/. The obtained intensity values were normalized to those of the wild-type strain, which were set to 1. The results were presented as average from four independent experiments.

### RNA extraction and RT-PCR.

Total cellular RNA was extracted from *A. oris* cells grown to mid-log phase and normalized to an OD_600_ of 1.0. Cell pellets were washed once with PBS and then frozen at −80°C. Then, the cells were suspended in RLT buffer and mechanically lysed using a bead beater. The RNeasy minikit (Qiagen) was used to extract nucleic acids from the samples. Off-column samples were treated with DNase (Qiagen), and then the RNeasy MinElute cleanup kit (Qiagen) was used to purify RNA.

For RT-PCR, cDNA was synthesized with Moloney murine leukemia virus reverse transcriptase (M-MLV RT) (Invitrogen). Parallel samples without the M-MLV RT enzyme were used as a control. The resulting cDNA samples were used in RT-PCR amplification with primers RT-lcpA-F and RT-lcpA-R (Table S2) combined in *Taq* RED master mix (Apex). Genomic DNA (gDNA) from the wild-type and Δ*lcpA* strains was used as control for specificity. The products were separated on a 1.5% agarose gel, stained, and imaged.

### Thermofluor assay.

The Thermofluor assay utilized SYPRO orange (Sigma), which binds to hydrophobic surfaces of proteins exposed during the unfolding process (21). eLcpA or its derivative (R149A and C179A/C365A) was prepared in 20 mM Tris-HCl, pH 8.0, to the final concentration of 5 mM. Reactions, the mixtures for which contained 45 μl of each protein solution mixed with 5 μl of 200× SYPRO orange solution, were performed in 96-well PCR plates using a Bio-Rad CFX real-time PCR system with the initial temperature set at 25°C. The fluorescence intensity was recorded every 30 s of 0.5°C increments until the final temperature of 99°C. The melting temperature (*T_m_*) of each protein determined using the Bio-Rad CFX program was obtained from three independent experiments performed in triplicate.

### Pyrophosphatase assay.

The pyrophosphatase activity of recombinant LcpA was determined according to a previously published protocol (9). Farnesyl pyrophosphate (FPP) at different concentrations (0 to 150 μM) was treated with recombinant LcpA (3 μM) in 20 mM Tris-HCl (pH 8.0) for 24 h at 30°C. Inorganic phosphate released from these reactions was detected by a phosphate fluorometric assay kit (MAK031; Sigma) according to the manufacturer’s instructions. Fluorescent phosphate signal was measured with a microplate reader (Tecan M1000) at the excitation wavelength of 535 nm and the emission wavelength of 587 nm. Phosphate standards were used to generate a standard curve, with samples without phosphate used as background fluorescence. The phosphate concentrations in the test samples were determined by linear regression analysis of the standard curve. The results were presented as an average from three independent experiments. Statistical analyses were performed with Prism GraphPad (version 5.04).

### Phosphotransfer assay.

GspA^LMM^ at 12 µM was incubated with 4 µM LcpA (WT or R149A) and 50 µM FPP in 20 mM Tris-HCl (pH 8.0) for 72 h at 30°C. After incubation, the protein samples were treated with hydrofluoric acid (HF), according to published protocols (25, 40–42), or mock treated. Briefly, protein samples were treated with 46% HF at 4°C for 18 h. After acid removal by vacuum evaporation, the protein samples were washed with 500 µl of deionized water followed by vacuum evaporation.

To analyze protein samples by 2-D electrophoresis, the samples were solubilized in sample/rehydration buffer (Bio-Rad) for 30 min at 25°C. For the first dimension, the protein samples were loaded onto IPG strips with a narrow, linear range (pH 3 to 6) using a Protean IEF system (Bio-Rad). After isoelectric focusing (IEF), the IPG strips were placed onto a 4 to 20% Criterion TGX IPG+1 gel (Bio-Rad) in overlay agarose (0.5% agarose in 1× Tris-glycine-SDS [TGS] buffer and 0.003% bromophenol blue) alongside a Precision Plus protein dual color standard (Bio-Rad). The proteins in the second dimension were then transferred to a PVDF membrane for subsequent immunoblotting with anti-GspA antibodies.

### NMR data collection and analysis.

All protein samples used for NMR experiments were concentrated and dialyzed into NMR buffer (50 mM Tris, pH 6.5, 100 mM NaCl, 5 mM MgCl_2_, 10% D_2_O). ^1^H-^15^N HSQC NMR spectra were recorded on a Bruker 500-MHz spectrometer at room temperature equipped with a triple resonance cryogenic probe. Initial ^15^N-eLcpA data were collected at 250 μM, with 128 scans and 256 points in the indirect nitrogen dimension. Scan number was adjusted to account for sample dilution during titration. Data were processed using NMRPipe (43), and spectra were analyzed in Sparky (44).

### Data availability.

The coordinates are deposited in the PDB under accession code 5V8C.
