# Erratum for Siegel et al., “Structure and Mechanism of LcpA, a Phosphotransferase That Mediates Glycosylation of a Gram-Positive Bacterial Cell Wall-Anchored Protein”

**DOI:** 10.1128/mBio.00617-19

**Published:** 2019-04-16

**Authors:** Sara D. Siegel, Brendan R. Amer, Chenggang Wu, Michael R. Sawaya, Jason E. Gosschalk, Robert T. Clubb, Hung Ton-That

**Affiliations:** a Department of Microbiology and Molecular Genetics, University of Texas Health Science Center, Houston, Texas, USA; b Department of Chemistry and Biochemistry and the UCLA-DOE Institute of Genomics and Proteomics, University of California, Los Angeles, California, USA; c Division of Oral Biology and Medicine, School of Dentistry, University of California, Los Angeles, California, USA

## ERRATUM

Volume 10, issue 1, e01580-18, 2019, https://doi.org/10.1128/mBio.01580-18. The following funding information should have been included:

