# Supplementary material for: Structure and Mechanism of LcpA, a Phosphotransferase That Mediates Glycosylation of a Gram-Positive Bacterial Cell Wall-Anchored Protein
Source: mBio. 2019 Feb 19;10(1):e01580-18. doi: 10.1128/mBio.01580-18 (PMC6381275; doi:10.1128/mBio.01580-18)
Supplement: FIG S1 [file mBio.01580-18-sf001.pdf]

```
AoLcpA       1 MDSTSH--------------------------------------------------------------------------
AoLcpB       1 MTQSLL--------------------------------------------------------------------------
AoLcpC       1 MIA-----------------------------------------------------------------------------
AoLcpD       1 VTPTDDSLPPSITPGSGGGRPQRRQRPIDAVNQRSRERGEASGGEAVAQPTDVAGGSPRRPSSPTARRPQRHSVLGRNDS
BsTagT       1 MEERSQ--------------------------------------------------------------------------
SpnCspA2     1 MGLTNR---------------------LNAT----SNYSEYSLSVA----------------------------------

AoLcpA       7 ---------------------DEHSAAPSEASE-ASD--ATGDPTDTSSPLPQR-----------------------RR-
AoLcpB       7 ---------------------PEPESSPIEKGD-AEAASSGADTTS----------------------------------
AoLcpC       4 -------------------------------------------------PVPQF-----------------------SKP
AoLcpD      81 DRPASAQSASVREGQQPPSVQPRRSSAPSQRGSHARAGSSAIPLEPDAGVGNGASEPERTQAMPARRGTYPVGSSAPRE-
BsTagT       7 --------------------------------------------------------------------------------
SpnCspA2    22 -------------------VLADSEIENVTQLT-SVTAPTGTDNE-----------------------------------

AoLcpA      39 QGRG-GTG-----V---KRWSRRRKIV-VGLST--AVMTTALAL--GADVAV--L--------------AHRPAR--VDI
AoLcpB      31 -ARG-AGS----VL---RRPGRGW-RI-ALLSV--LAIFLVLTLATGGLALWVRHS------------IASGIEFI-ADP
AoLcpC      12 HARH-SS----------KELGRRLARR-VGLSL--LAVTIFVVS--AAGFAW--HN------------IQSRITWFNIDS
AoLcpD     160 PAAG-GPSLRPERPRRPQRSRPRWRRI-VGWTV--VVIVVALALI-AARIAWLWND------------VSSQLHRV--DA
BsTagT       7 -RRK---------K---KRKLKKWVKVVAGLMA--FLVIAAGSV--GA-YAFVKLN------------NASKEAHV--SL
SpnCspA2    47 NIQKLLADIK---------SSQNTDLT-VNQSSSYLAAYKSLIA--GETKAIVLNSVFENIIELEYPDYASKIKKI--YT

AoLcpA      87 A-----M----PTTSSPTAGETWLILGTDSRATVPGDQNRYGTTQ-EVEGSRADVIAL--VRPSQEGVTIINLPRDLTIN
AoLcpB      85 FAGIPARAPQQKVAAGEEPAVNILVLGTDSRTSASDP------SQWKEGAQRTDAIMIVQVSGDRKTVSVMSIPRDSWVE
AoLcpC      62 LLSEEDRPGTKPPDSYEGRAVNLLILGTDSRAGSNNVD-GSQGDD-EVSVARSDTALVMHISADRKRVDAVSIPRDTLVD
AoLcpD     221 LSGA-----------ADTPGETWLIVGSDARGGAVQ--------D-ETEGARADSVMLLH-KADNGQTSLTSLPRDTYVD
BsTagT      55 ARGEQSVKRIKEFDPG-KDSFSVLLLGIDAREKNG---------E-TVDQARSDANVLVTFNRKEKTAKMLSIPRDAYVN
SpnCspA2   113 ---KGFTKKVEAPKTSKNQSFNIYVSGIDTYGPI-------------SSVSRSDVNILMTVNRDTKKILLTTTPRDAYVP

AoLcpA     155 SKGME---L-----------DRLATT--------------YVPGPQNTVNALCTGLGIPTTHLVTIDMAQFATIIDSLGG
AoLcpB     159 IPGHG---Q-----------GKINAAYS-------------YGGPSLTIHTVENLTGIHIDHFAVANFESFVALTDEIGG
AoLcpC     140 IPECT---TLDGGKTEASEDAPFNSAFAKGAGSSSDDKKAVASGAACTLKTVETLTNVRIDDFIVVDFTGLSKVVDSLGG
AoLcpD     280 IPEYG---E-----------NKINAAYS-------------FGGPKLLVQTVEKLSGLTVDHYVEVGMTGVSQMVDAVGG
BsTagT     124 IPGHG---Y-----------DKFTHAHA-------------YGGVDLTVKTVEEMLDIPVDYVVESNFTAFEDVVNELNG
SpnCspA2   177 IADGGNNQK-----------DKLTHAG--------------IYGVDSSIHTLENLYGVDINYYVRLNFTSFLKMIDLLGG

AoLcpA     207 IEVDVPEPV-----RDA-YTGL--NLSSA-GRHRLSGIDALALVRSRHPEILRDGRWVTMSQADG-AQRRSQSTATVMQA
AoLcpB     212 VRINLKTP-------QT-LAGK--EL-GA-GAQVLDGQQALAYTRERSS------------LPNG-DFDRVKRQQTWMRS
AoLcpC     217 VHVQVDEAI-----DDSEYTGF--KL-AE-GCQKLDGENALQYARVRHG------------VSDGSDLSRITRQQNLMRA
AoLcpD     333 VNVCLDYDV-----ADE-DSGLVWNT-SQGTCQTVDGTKALAYSRMRKS------------DPNG-DVGRGQRQRAVISA
BsTagT     177 VKVTVKSDKVIQQIKKDTKGKV--VL-QK-GTHTLDGEEALAYVRTRKA--------------DS-DLLRGQRQMEVLSA
SpnCspA2   232 VDVHNDQEF-----SAL-HGKF--HF-PV-GNVHLDSEQALGFVRERYS------------LADG-DRDRGRNQQKVIVA

AoLcpA     277 VLSAIGQKA--SNPVS-LHQLAHTVAGNITLDSGTGLSDLAALGRSASKARRAGATT---IIDLPTGPR---DES-II-V
AoLcpB     267 IVSRVLTNGTMSSPTA-LYSFLKTASRTVAVDESFTLNQMQSLALETRH-LHSNDIA---FMTVPTAGTGTSTDGQSI-V
AoLcpC     276 MASKALSSSLLTQSGF-LTSTLETLTTSERIGQ---IGNLSGLAYSIQG-VGIDKIN---FVTMPNEPA---ADP-NQ-V
AoLcpD     393 VVSKAAAPSTAFSFSR-QDALVDAGTNALTVDRSAGTMSIAQMVLAFRS-ASGSGLTGAPPIEDPAYSP---EDANIGET
BsTagT     238 IIDKSKSLSSIPAYDDIVDTMGQNLKMNLSLKDAIGLFPFI---TSLKS-VE--------SIQLTGYDY---EPAGVY-Y
SpnCspA2   289 ILQKLTSTEALKNYSTIINSLQDSIQTNVPLETMINLVNAQLESGGNYK-VNSQDLKGTGRMDLPSYAM---PDSNLY-V

AoLcpA     346 SPNQE-SR-DLL-------------------------------------------------ARYGYSPKTC----RPAG-
AoLcpB     341 TLDADADT-PLFNAFAEDRVSTY-------------------------------------------------LTEHPDAV
AoLcpC     343 VPSEG-AK-KVWKALEEDKPVPSDTSAPASSDSSATTNDSSSQSTDESAETSDPSQATPTPAPTTQAPNASTPQQQPAAK
AoLcpD     468 VLLQDTTAPNFFSKLRDG--------------------------------------------------------------
BsTagT     302 FKLNQQKLQEVKKELQNDLGV-----------------------------------------------------------
SpnCspA2   364 MEIDDSSL-AVVKAAIQDV-------------------------------------------------MEG---------

AoLcpA     370 ---------A
AoLcpB     371 ELLPATV--N
AoLcpC     421 PTTPACP--A
AoLcpD     486 KLTAADFNQS
BsTagT         ----------
SpnCspA2   385 RKLAAALE--
```

Figure S1: Siegel, Amer et al.
