# Supplementary figures and images for: Structure and Mechanism of LcpA, a Phosphotransferase That Mediates Glycosylation of a Gram-Positive Bacterial Cell Wall-Anchored Protein
Source: mBio. 2019 Feb 19;10(1):e01580-18. doi: 10.1128/mBio.01580-18 (PMC6381275; doi:10.1128/mBio.01580-18)

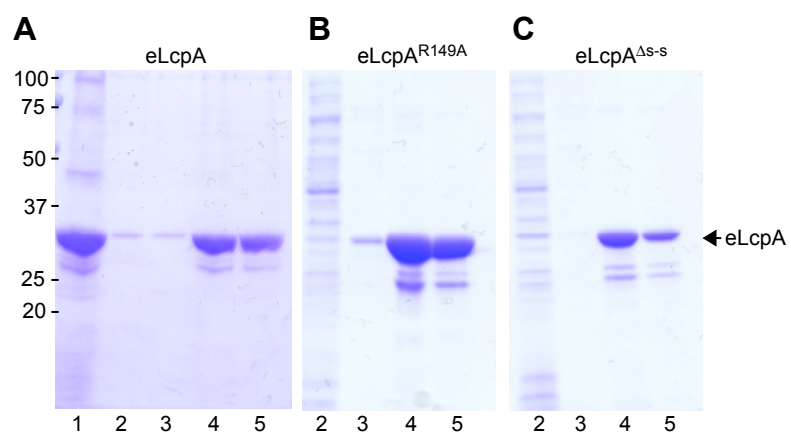

Figure S2: Siegel *et al.*

Supplement: FIG S2 [file mBio.01580-18-sf002.pdf]

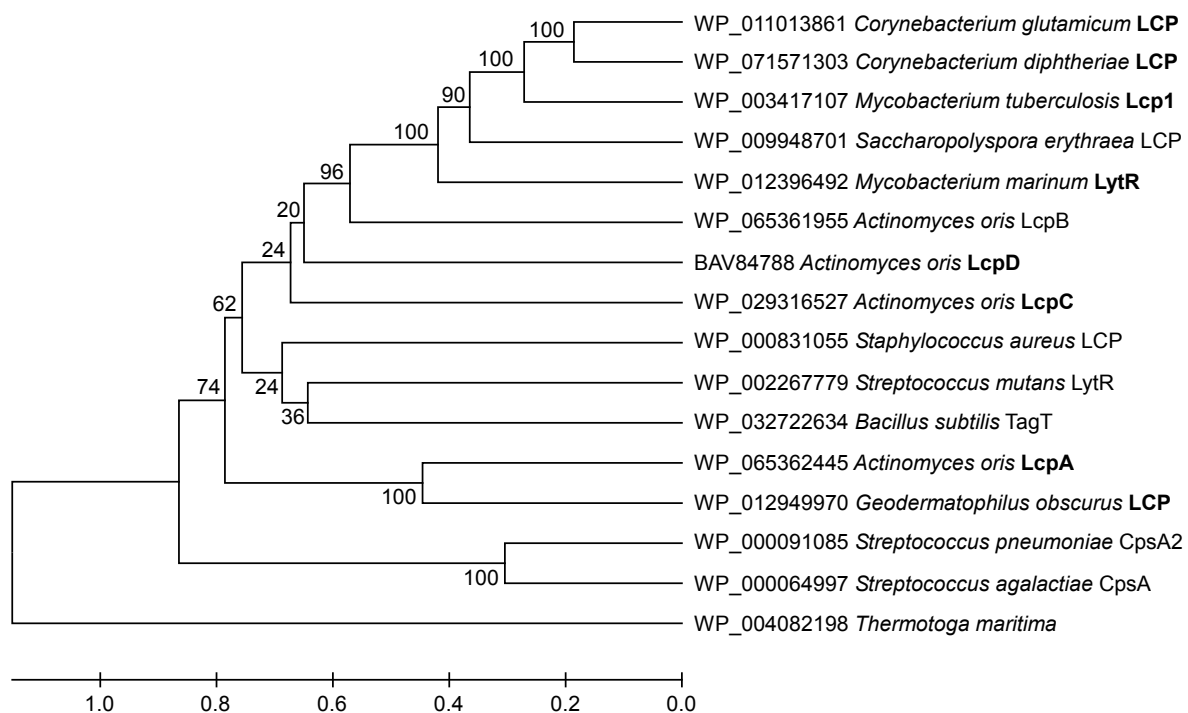

Figure S3: Siegel *et. al.*

Supplement: FIG S3 [file mBio.01580-18-sf003.pdf]

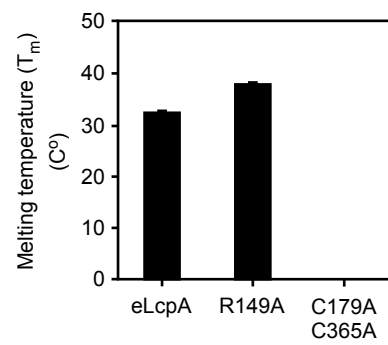

Figure S4: Siegel et al.

Supplement: FIG S4 [file mBio.01580-18-sf004.pdf]
