# Supplementary material for: Structure and Mechanism of LcpA, a Phosphotransferase That Mediates Glycosylation of a Gram-Positive Bacterial Cell Wall-Anchored Protein
Source: mBio. 2019 Feb 19;10(1):e01580-18. doi: 10.1128/mBio.01580-18 (PMC6381275; doi:10.1128/mBio.01580-18)
Supplement: TABLE S1 [file mBio.01580-18-st001.pdf]

Supplemental Table S1: Bacterial strains and plasmids

Strain or Plasmid	Description	Reference
Strain		
A. oris MG-1	Parental strain; wild-type	(2)
A. oris CW1	Δ*galK*; an isogenic derivative of MG-1	(3)
A. oris WU51	Δ*gspA;* an isogenic derivative of CW1	(4)
A. oris WU72	Δ*lcpA;* an isogenic derivative of CW1	(4)
A. oris D0299	Δ*lcpB;* an isogenic derivative of CW1	This study
A. oris D1578	Δ*lcpD;* an isogenic derivative of CW1	This study
A. oris lcpΔ3	Δ*lcpA*Δ*lcpB*Δ*lcpD*	This study
A. oris MR108	ΔVKOR; an isogenic derivative of CW1	(5)
E. coli C3209/ Shuffle®	Derived from *E. coli* K12 parental strains, engineered to form disulfide bonds in the cytoplasm; expresses T7 RNAP.	(6)
Plasmid		
pCWU2	*E. coli* cloning vector, *Actinomyces* suicide vector, KmR	
pCWU10	*Actinomyces*/*E. coli* shuttle vector, KmR	(7)
pMCSG7	Expression vector for protein purification	(8)
pKO-0299	pCWU2 derived KO plasmid Δ*lcpB*	This study
pKO-1578	pCWU2 derived KO plasmid Δ*lcpD*	This study
eLcpA	pMCSG7 expressing LcpA(78-370)	This study
eLcpA(R149A)	rLcpA with the mutation R149A	This study
eLcpA(Δs-s)	rLcpA with the mutation C179A/C365A	This study
pLcpA	pCWu10 expressing LCP from native promoter	This study
pLcpA(R128A)	pLcpA with the mutation R128A	This study
pLcpA(R149A)	pLcpA with the mutation R149A	This study
pLcpA(R266A)	pLcpA with the mutation R266A	This study
pLcpA(C365A)	pLcpA with the mutation C365A	This study
pLcpA(C179/365A)	pLcpA with the mutation C179/365A	This study
pVKOR-2HA	pCWU10 expressing VKOR with double C-terminal influencza hemagglutinin (HA) tag	(5)
pGspA$_{\Delta cws}$-H6	pCWU10 expressing GspA lacking the cell wall sorting signal (CWS) with Hisx6 tag	(7)
rGspA	pMCSG7 expressing GspA(31-286)	This study

Supplemental References

1. Sievers F, Wilm A, Dineen D, Gibson TJ, Karplus K, Li W, Lopez R, McWilliam H, Remmert M, Soding J, Thompson JD, Higgins DG. 2011. Fast, scalable generation of high-quality protein multiple sequence alignments using Clustal Omega. Mol Syst Biol 7:539.
2. Mishra A, Das A, Cisar JO, Ton-That H. 2007. Sortase-Catalyzed Assembly of Distinct Heteromeric Fimbriae in *Actinomyces naeslundii*. J Bacteriol 189:3156-65.
3. Mishra A, Wu C, Yang J, Cisar JO, Das A, Ton-That H. 2010. The *Actinomyces oris* type 2 fimbrial shaft FimA mediates co-aggregation with oral streptococci, adherence to red blood cells and biofilm development. Mol Microbiol 77 841–854.
4. Wu C, Huang IH, Chang C, Reardon-Robinson ME, Das A, Ton-That H. 2014. Lethality of sortase depletion in *Actinomyces oris* caused by excessive membrane accumulation of a surface glycoprotein. Mol Microbiol 94:1227-41.
5. Luong TT, Reardon-Robinson ME, Siegel SD, Ton-That H. 2017. Reoxidation of the Thiol-Disulfide Oxidoreductase MdbA by a Bacterial Vitamin K Epoxide Reductase in the Biofilm-Forming Actinobacterium *Actinomyces oris*. J Bacteriol 199:e00817-16.
6. Bessette PH, Aslund F, Beckwith J, Georgiou G. 1999. Efficient folding of proteins with multiple disulfide bonds in the *Escherichia coli* cytoplasm. Proc Natl Acad Sci U S A 96:13703-8.
7. Siegel SD, Wu C, Ton-That H. 2016. A Type I Signal Peptidase Is Required for Pilus Assembly in the Gram-Positive, Biofilm-Forming Bacterium *Actinomyces oris*. J Bacteriol 198:2064-73.
8. Donnelly MI, Zhou M, Millard CS, Clancy S, Stols L, Eschenfeldt WH, Collart FR, Joachimiak A. 2006. An expression vector tailored for large-scale, high-throughput purification of recombinant proteins. Protein Expr Purif 47:446-54.
