# Supplementary material for: Structure and Mechanism of LcpA, a Phosphotransferase That Mediates Glycosylation of a Gram-Positive Bacterial Cell Wall-Anchored Protein
Source: mBio. 2019 Feb 19;10(1):e01580-18. doi: 10.1128/mBio.01580-18 (PMC6381275; doi:10.1128/mBio.01580-18)
Supplement: TABLE S2 [file mBio.01580-18-st002.pdf]

Supporting Table S2: Primers used in this study

Primer	Sequence[a]	Application
D0299up-F	GGCGGGTACCGTGACGAGCAGCGCCGCTGCGCT	Δ*lcpB*
D0299up-R	GGCGTCTAGAGCGAGGTCGTGTCGGCCCCTGACGAG	Δ*lcpB*
D0299dn-F	GGCGTCTAGAGTCACGCTCGACGCCGACGCGGACAC	Δ*lcpB*
D0299dn-R	GGCGGAATTCAGCTCCTCAACCGCCTCGGGCAC	Δ*lcpB*
D1578up-F	GGCGGGTACCTCTCCTACGTCCTGGAGAAGACGA	Δ*lcpD*
D1578up-R	GGCGTCTAGAGCGATCATAGGGAACGAGACTGCTA	Δ*lcpD*
D1578dn-F	GGCGTCTAGAGGCCAACATCGGCGAGACGGTACTG	Δ*lcpD*
D1578dn-R	GGCGGAATTCGCGCAGATGTTGCGCACCCTCACGT	Δ*lcpD*
eLcpA-F	TACTTCCAATCCAATGCAGCTCATCGCCCTGCACGCG	eLcpA
eLcpA-R	TTATCCACTTCCAATGTTACTAGGCGCCCGCTGGCC	eLcpA
LcpA-F	GGATCCTCGCCTCCTTCCAGTCTGACTGG	pLcpA
LcpA-R	GAATTCCCTCGGGGTCTCTCCGGCGAGTG	pLcpA
LcpA(R128A)-F	GCCGCCGATGTCATCGCCCTGGTACGC	SDM[b] LcpA
LcpA(R128A)-R	GGAACCCTCCACCTCCTGAGTG	SDM LcpA
LcpA(R149A)-F	GCGGACCTGACCATCAACAGCAAGG	SDM LcpA
LcpA(R149A)-R	GGGCAGGTTGATGATGGTGACTC	SDM LcpA
LcpA(R266A)-F	GCCAGCCAGTCCACGGCCACCGTG	SDM LcpA
LcpA(R266A)-R	GCGTTGGGCGCCATCGGCC	SDM LcpA
LcpA(C179A)-F	GCCACCGGGCTCGGAATCCCCAC	SDM LcpA
LcpA(C179A)-R	CAGGGCGTTGACCGTGTTCTGAGG	SDM LcpA
LcpA(C365A)-F	CGCACGGCCAGCGGGCGCCTAG	SDM LcpA
LcpA(C365A)-R	GTTTTGGGACTGTACCCATAGCGGGC	SDM LcpA
RT-lcpA-F	CGGCAGATGGGTGACCATGAGC	RT-PCR[c]
RT-lcpA-R	CAGTGCGGCCAGGTCGCTGAG	RT-PCR
rGspA-F	TACTTCCAATCCAATGCATCCCTCGCCTTCAAGATCGCCG	rGspA
rGspA-R	TTATCCACTTCCAATGTTACTTGCCGGAGGTGGAGGCCGC	rGspA

[a] Restriction sites are underlined.

[b] For *s*ite-*d*irected *m*utagenesis

[c] For reverse transcription polymerase chain reactions
